# Can we rely on delaying the uptake of sugar consumption in the prevention of early childhood caries?

**DOI:** 10.1038/s41432-025-01125-8

**Published:** 2025-02-26

**Authors:** Orion O’Brien, Greig D. Taylor

**Affiliations:** Newcastle University, School of Dental Sciences, Newcastle upon Tyne, UK

**Keywords:** Paediatric dentistry, Nutrition and diet in dentistry

## Abstract

**A Commentary on:**

**Feldens C A, de Barros Coelho E M R, Vítolo M R, Rogrigues P H, Kramer P F, Peres K G**

Effectiveness of a sugar consumption prevention programme in the first year of life on the occurrence of early childhood caries: a multicentric randomized trial in Brazil. *Caries Res* 2024; 10.1159/000541028.

**Study design:**

A multi-centre randomised controlled trial was carried out in Brazil. The intervention group received nutritional counselling based on dietary guidelines produced by UNICEF. The control group received standard dietary and maternal counselling provided by the hospital provider. Randomisation of participants was completed through a computer-based system with an investigator blinded to the recruitment process. Patients were followed up monthly using a combination of phone calls and home visits.

**Sample:**

Pregnant women were recruited from four hospitals, across three state capitals, participating in the Baby Friendly Hospital Initiative. Mothers were to be at least 18 years of age, testing negative for HIV/HTLV1, and had an uncomplicated pregnancy. Their newborns had to have a gestational age of more than 37 weeks and weigh more than 2.5 kg. A newborn with increased hospital stays due to infection or neonatal conditions which affected breastfeeding were not included. Sample size calculation was undertaken.

**Data analysis:**

Baseline data was collected. At 6- and 12-month intervals, a combination of validated questionnaires, including 24-hour recalls, and interviews were used to assess the diet. Oral health assessments were carried out by a blinded, trained and calibrated paediatric dentist. The primary outcome was a reduction in ECC. Effect measures (relative risk [RR]) were calculated to determine the effect of the intervention on not consuming sugar at 6 months and on the mean number of sugary items consumed at 12 months.

**Results:**

Baseline demographic data were similar, with no significant differences noted, between the intervention and control groups. The probability of not consuming sugar in the first 6-months was 2.4 times less in the intervention group relative to the control group (*p* = 0.016). ECC was diagnosed in 17.4% of the whole sample; however, no significant difference was noted between groups at any time point (*p* = 0.281).

**Conclusions:**

Increased intervention to the mother in the first 6-months of life was effective at reducing the amount of sugar intake. However, this did not lead to a statistically significant reduction in ECC.

## GRADE Rating:



## Commentary

The authors present a well-designed randomised clinical trial to investigate the impact of a tailored sugar prevention programme on the development of early childhood caries (ECC)^1^.

The study design is appropriate for the intended research question. It is reassuring that a power calculation was performed to determine sample size although this may have been a post-hoc sample size calculation. Randomisation and allocation to the intervention and control groups were completed effectively through the use of a computerised system and without bias, and the investigator responsible for this was blinded to the recruitment process. There was a large drop-out rate which an intention-to-treat analysis accounted for. However, minimal information was provided on how missing data was dealt with.

The large drop-out rate reduced the sample size. Fortuitously, the drop-out rate was similar for both groups. The authors acknowledge this limitation and attribute this to change in participant address and/or refusal to participate. Alternatively, it could be a disinterest in the study due to the participant burden of monthly phone calls/home visits on parents who are caring for a young child. Engagement with primary dental care or ante-natal team was not been recorded and refusal to participate may also be due to mother and child receiving risk-based prevention from another provider, negating the need for their involvement.

Given the nature of the intervention, dietary counsellors were not blinded. In contrast, data collection on diet (by interviewers) and oral health (by a paediatric dentist) were undertaken by trained, calibrated and blinded individuals. Appropriate inter- and intra-rater scores were provided. Good internal validity to the study was apparent as the follow-up process was robustly reported, reassuring the reader that the methods were undertaken in a consistent and robust manner. Unfortunately, the main outcome was the impact on ECC. ECC is described up to the age of three^2^, thus the short follow-up period limits the full extent of ECC rates in both cohorts. A longer follow-up period of the trial would have been important to fully establish the impact.

The more intensive counselling seems to have been effective at preventing early introduction to dietary and additional sugars to children at both 6-months and 12-months, expressed through the sugar consumption index. The validity of this outcome is however questionable. The use of limited questionnaires identifying only food and drink with added sugar, alongside 24-hour recall, is non-specific and does not provide longitudinal information of the children’s sugar consumption. The intervention group also received advice with a focus on avoiding added sugars. The use of a questionnaire that quantified foods with added sugar to record the sugar consumption index could result in acquiescence bias in the intervention group, with them only reporting the foods that are seen as correct. In addition, the resource and cost required for the intervention counselling has not been accounted for. A simple cost-analysis, or fuller economic evaluation would have supported whether the intervention is cost-effective or not. Having this information would be of benefit to policymakers who consider how best to allocate fixed and scarce resources.

No significant difference was found between the two groups for the occurrence of ECC and caries-affected teeth. The intervention group however did report a lower percentage of those diagnosed with ECC (19.6%) when compared with the control group (14.7%). The intervention group also had a lower maximum number of teeth affected by caries (4) versus the control group (6). This would suggest that an intervention is better than none, but no significant difference was seen between the two. Precision was effectively reported with confidence intervals and no harms or unintended effects of the intervention were made clear by the authors. The programme targeted only diet but did not account for other factors like fluoride use or oral hygiene which contribute to caries disease progression. There was also no record of how the children have been fed. The most common pattern of ECC is that of ‘bottle caries’ which is the introduction of a sugary beverage via a bottle frequently^3^. This frequent consumption of a sugary drink is often the prime aetiological factor in ECC^3^ but there are no details of what the children were most commonly eating or drinking. Furthermore, there was no data on the breastfeeding habits of the mothers. Despite varied evidence of the impact of breastfeeding on ECC, breastfeeding up to the first year could have an impact on the occurrence of ECC due to the reported protective effect^4^. No oral hygiene instruction was included in the intervention and both groups presented with similar plaque scores at their 12-month review. With no mention of outside involvement that may be providing further advice, we cannot account for this confounding factor. There has also been no reporting on the brushing habits or use of fluoride toothpaste. Without consideration of these other factors that are known to prevent dental caries, it is not possible to conclude that the programme had no impact on the occurrence of ECC. Accounting for these various confounding factors could have been dealt with in a regression analysis.

While the prevention of sugar intake is a positive, the programme had no impact on ECC and it is unclear whether it is cost-effective. Furthermore, advanced prevention including diet advice can be provided by general dentists and supported by guidance to support the dental practitioner in providing targeted advice^5,6^.

The results of this study are perhaps best used in the development of other studies/programmes to target ECC. It highlights that dietary counselling alone is insufficient to influence ECC and suggests that such initiatives should be part of a broader preventive programme. Overall, while the study design was sound, its conclusions are confounded by the lack of consideration of other preventative factors.

Practice Points

- Sugar consumption prevention programmes can reduce sugar intake in the first year of life.
- Diet modification is not the only factor to address when trying to prevent ECC.
